# High Temperature, High Ambient CO_2_ Affect the Interactions between Three Positive-Sense RNA Viruses and a Compatible Host Differentially, but not Their Silencing Suppression Efficiencies

**DOI:** 10.1371/journal.pone.0136062

**Published:** 2015-08-27

**Authors:** Francisco J. Del Toro, Emmanuel Aguilar, Francisco J. Hernández-Walias, Francisco Tenllado, Bong-Nam Chung, Tomas Canto

**Affiliations:** 1 Departamento de Biología Medioambiental, Centro de Investigaciones Biológicas, CSIC, Ramiro de Maeztu 9, 28040, Madrid, Spain; 2 National Institute of Horticultural & Herbal Science, Agricultural Research Center for Climate Change, 281, Ayeon-ro, 690–150, Jeju, Jeju Island, Republic of Korea; University of Basel, SWITZERLAND

## Abstract

We compared infection of *Nicotiana benthamiana* plants by the positive-sense RNA viruses *Cucumber mosaic virus* (CMV), *Potato virus Y* (PVY), and by a *Potato virus X* (PVX) vector, the latter either unaltered or expressing the CMV 2b protein or the PVY HCPro suppressors of silencing, at 25°C vs. 30°C, or at standard (~401 parts per million, ppm) vs. elevated (970 ppm) CO_2_ levels. We also assessed the activities of their suppressors of silencing under those conditions. We found that at 30°C, accumulation of the CMV isolate and infection symptoms remained comparable to those at 25°C, whereas accumulation of the PVY isolate and those of the three PVX constructs decreased markedly, even when expressing the heterologous suppressors 2b or HCPro, and plants had either very attenuated or no symptoms. Under elevated CO_2_ plants grew larger, but contained less total protein/unit of leaf area. In contrast to temperature, infection symptoms remained unaltered for the five viruses at elevated CO_2_ levels, but viral titers in leaf disks as a proportion of the total protein content increased in all cases, markedly for CMV, and less so for PVY and the PVX constructs. Despite these differences, we found that neither high temperature nor elevated CO_2_ prevented efficient suppression of silencing by their viral suppressors in agropatch assays. Our results suggest that the strength of antiviral silencing at high temperature or CO_2_ levels, or those of the viral suppressors that counteract it, may not be the main determinants of the observed infection outcomes.

## Introduction

Anthropogenic releases of CO_2_ and of other gases very likely increase global temperatures and may lead to changes in local climates (http://www.ipcc.ch/). In this context, modern agriculture becomes exposed to losses from outbreaks of viral diseases that derive from increased global trade and warmer environments [1]. Among plant viruses, single-stranded, positive-sense RNA (ssRNA+) viruses are taxonomically the largest group and some, such as cucumoviruses and potyviruses cause relevant economic losses [2]. The outcomes of any virus/plant compatible infection also depend on the physiological state that environment parameters, such as temperature or CO_2_ levels induce in the host, which provoke changes affecting plant growth rate and shape, carbon:nitrogen ratios, and in the molecular pathways that modulate responses to both, external biotic or abiotic factors [3, 4, 5, 6, 7]. Knowledge gained on compatible viral infections when these parameters alter, and on the molecular mechanisms underpinning them is therefore of interest. With regard to elevated environment CO_2_ levels our knowledge on compatible plant-virus interactions it is still limited: CO_2_ levels of 1000 parts per million (ppm) reduced *Potato virus Y* (PVY) titers in tobacco fresh weight samples [4], and 750 ppm alleviated the damage this virus caused in the same host [8]. The latter levels also decreased both symptom severity and virus titers in the infection of tomato by the geminivirus *Tomato yellow leaf curl virus* and also of *Tobacco mosaic virus* in the same plant [3, 7] respectively, probably because of alterations in the levels of salicylic acid (SA) and jasmonic acid (JA). In this regard, elevated CO_2_ decreased JA but increased SA levels in uninfected arabidopsis plants [9]. With regard to temperature, it is known from early works that increases in temperature can lead to weaker infection symptoms (heat masking effect) [2]. Interestingly, more recent reports also indicate that a main defense of plants against viruses, small interfering RNA (siRNA)-mediated gene silencing, increases in strength with temperature. In a number of compatible infections, weaker symptoms correlated with slight-to-marked decreases in the accumulation of the corresponding viruses, and with an increase in the ratios of small RNAs to viral sequences: in ssRNA+ viruses *Turnip crinkle carmovirus* and *Cymbidium ringspot tombusvirus*, when increasing temperatures from 21 to 27°C in *Nicotiana benthamiana* [10, 11], for the negative ssRNA *Citrus psorosis virus* in sweet orange, from a range of 26/18°C (day/night) to 32/26°C [12], or for several DNA geminiviruses from 25 to 30°C in cassava and *N*. *benthamiana* [13]. The molecular basis for this effect of temperature on antiviral gene silencing is being investigated, but could be related to increased biological activities at higher temperatures of some protein components of its molecular pathways. This was proposed for host RdRp and/or Dicer-like (DCL) activities [13] and was demonstrated for DCL 2, which at 26°C (vs. 20°C) increases synthesis of 22-nucleotide small RNAs to positive-sense RNA virus *Turnip crinkle virus* sequences in arabidopsis plants [14]. However, high temperatures (30 vs. 22°C) reduced the silencing induced on endogenous genes by homologous integrated sense transgenes in arabidopsis plants, possibly as the levels of *Suppressor of gene silencing 3* gene product (SGS3) involved in formation of secondary siRNAs, diminished at 30°C in this host [15]. SiRNA-based silencing strength against mRNAs expressed from transient agroinfiltrated T-DNAs, as well as against integrated antisense transgenes has also been shown to increase gradually with temperature, from 15 to 24°C in *N*. *benthamiana* [11], likely for the same underlying reasons than for viruses. It has been hypothesized that heat masking (decreased viral titers and symptoms) at high temperature derive from enhanced antiviral gene silencing defenses overcoming the counteracting activities of virus-encoded suppressors of silencing [10, 11, 13].

On the virus side, the effects of either temperature or CO_2_ levels on the biological activities of the viral suppressors that neutralize the host antiviral silencing defenses are little known. Recently, two viral suppressors, HCPro from PVY and the 2b protein from the cucumovirus *Cucumber mosaic virus* (CMV), both ssRNA+ viruses were shown to suppress in *N*. *benthamiana* agroinfiltration patch assays the partial silencing of a green fluorescent protein (GFP) reporter at high temperature (30°C) [16].

Processes other than RNA silencing also intervene in defending plants against viruses and could be susceptible to temperature or CO_2_ levels: degradation of viral factors through proteasome or autophagy, or elicitation of generic responses by phytohormones, such as SA or JA, or non-sense mediated decay [17, 18, 19, 20, 21, 22]. In addition, plant viruses must carry out processes involving viral and host components to successfully infect the plant, disperse and complete their infectious cycles. Current knowledge is limited on the effects of temperature, CO_2_ levels on any of these processes.

In this work we have characterized infection of *N*. *benthamiana* plants with five ssRNA+ viruses, at 25 vs. 30°C, or at standard (st) vs. potential end-of-the-century atmospheric CO_2_ levels [401 vs. 970 parts per million (ppm); http://www.ipcc.ch]. We found that alterations in severity of symptoms and virus titers were characteristic for each individual virus, but also that their encoded suppressors of silencing could prevent efficiently the host silencing response under those same conditions. These results suggest that the strength of antiviral silencing by the host or those of the viral suppressors that neutralize it cannot explain the differences in the outcomes of infection observed in these environment conditions, and therefore that other processes relevant to the viral cycle must be more determinant.

## Materials and Methods

### Plants and viruses

Plants: *N*. *benthamiana* plants were used, as they become systemically infected by the five viruses/virus vectors used in this study and are also amenable to transient gene expression by agroinfiltration. Transgenic *N*. *benthamiana* [23] expressing an inverted repeat fragment of its *RNA-dependent RNA polymerase 6 (RDR6*) gene that reduced the levels of the endogenous transcript mRNA to 4% of that found in non-silenced plants was also used, kindly provided by Prof. D. C. Baulcombe (University of Cambridge, UK). Viruses: we used an aphid transmissible PVY isolate (Scottish ordinary variety PVY-O, Scottish Agricultural Science Agency) from which the P1-6x-HCPro sequence contained in the binary constructs P1-6x-HCPro and PVX-P1-6x-HCPro [24, 25], respectively, and described below derive. We used a cloned CMV isolate, strain Fny, obtained by inoculating combined in vitro transcripts of full-length infectious clones of viral RNAs 1, 2, and 3 [24]. We also used three PVX vectors that were expressed from binary constructs (see below).

### Binary constructs for agroinfiltration

A GFP reporter and viral proteins were transiently expressed in plants from caulimovirus 35SP-driven, pROK2-based binary vectors: a vector expressing a free GFP reporter described in [26]; the binary vector expressing PVY P1-6x-HCPro (construct P1-6x-HCPro), described in [27]. The binary vector that expressed 2b protein with six histidines fused at its N- terminus, described by in [16].

Binary vector pgR107 kindly provided by Prof. D. C. Baulcombe group (University of Cambridge, UK) was used to express a modified infectious PVX that contains an additional CP promoter and a polylinker for the insertion and expression of foreign genes [28]. PVX expressing PVY P1-6x-HCPro was described in [27] and PVX expressing Fny CMV 2b protein with 6 histidines fused at its N terminus and an HA peptide at its C- terminus was described in [16].

### Delivery and expression of genes in plants by agroinfiltration, and local suppression of silencing in agropatch assays

For transient expression of proteins in plants by agroinfiltration, the corresponding binary constructs were transferred to non-oncogenic *Agrobacterium tumefaciens* strain C58C1, grown and infiltrated as described [16]. In the case of infectious PVX constructs, the binaries expressing them were expressed from *A*. *tumefaciens* strain GV3101 already containing a complementing pJIC SA_Rep plasmid [28].

In silencing suppression assays (agropatch assays), the free *GFP* reporter was co-expressed transiently in a *N*. *benthamiana* leaf patch, either with an empty binary vector, or with another vector expressing a protein to be tested for suppression of silencing activity. Leaves were then illuminated at 72 hours post infiltration (hpi) with a Blak Ray long wave UV lamp (UVP, Upland, CA, USA) to visualize and photograph the fluorescence derived from the transiently expressed free eGFP, and infiltrated patch disks were collected for protein analysis and quantification by western blot as described [26].

Agroinfiltrations were performed at lab room temperature (around 25°C). Immediately after infiltrations (no more than 15 minutes on the bench) plants were transferred to controlled plant growth chambers. In those cases where transient expression was to take place at higher temperatures (30°C), plants were maintained during the first 24 hpi at 25°C to allow for the agrobacterium-mediated T-DNA transfer into plant tissues to take place, as described [16].

### Plant inoculations and growing conditions

*N*. *benthamiana* plants were kept for the duration of the experiment in controlled growth chambers, with 16/8 hour day/night photoperiod and ~2500 lux of daylight intensity. Three environmental conditions were used: a) plants grown at 25°C and st CO_2_ partial pressure (~401 ppm): b) plants grown at 30°C and st CO_2_ levels; c) plants grown at 25°C and high CO_2_ partial pressure (~970 ppm). In c, plants were kept for the 7 days previous to viral challenge at 25°C and ~970 ppm, to allow them to adapt to the high CO_2_ environment.

Carborundum-dusted plants were inoculated with extracts at 10% (w/v) from PVY- or CMV-infected *N*. *benthamiana* plants, made in phosphate buffered saline, pH 6.8. For each of these two viruses, a single extract, aliquoted and kept at -80°C was used in all different experiments. For PVX constructs, plants were agroinoculated with agrobacterium containing the appropriate binary constructs as described in the previous section. In all cases, at the time of viral challenge, plants were 4–5 weeks old. Seven days after the challenge, leaf disks were collected for viral and host protein and nucleic acid assessments from the upper expanding leaves. Each type of experiment was performed at least twice

### Analysis of viral proteins by SDS-PAGE plus western blot and densitometry

For agroptach assays, agroinfiltrated leaf disks of around 25 mm in diameter (0.05 to 0.1 μg of fresh leaf tissue) excised from the leaf with a borer were used. For systemic plant tissue infected with viruses, 3 combined leaf disks/plant of 9.7 mm diameter were taken from the upper expanding leaves. In both cases, disks were excised from the leaf with a cylindrical borer. Total proteins were extracted by grounding disks in nitrogen with a pestle, and adding 400 μl of extraction buffer/0.05 g of leaf tissue (0.1 M Tris-HCl PH 8, 10 mM EDTA, 0.1 M LiCl, 1% β-mercaptoethanol and 1% SDS). Samples were boiled, and fractionated in 10% (for HCPro detection) or 15% (for GFP, 2b and viral CPs) SDS-PAGE gels. Gels were wet-blotted onto Hybond-P PVDF membranes (Amersham, GE Healthcare, Buckinghamshire, UK). Antisera used for detection of GFP, or of 2b protein bands were already described [26]. Detection of HCPro tagged with six histidines was performed using a mouse monoclonal antibody to PVY HCPro (Ab 1A11) [29]. For the detection of PVX CP a commercial rabbit antibody was used (No. 070375/500; Loewe Biochemica GmbH, Germany). For the detection of CMV CP we used a home-made rabbit polyclonal antiserum. Blotted proteins were detected using commercial secondary antibodies and SigmaFast BCIP/NBT substrate tablets (SIGMA Aldrich, Saint Louis, Missouri, USA). Densitometric analysis of blotted protein bands were performed using ImageJ (image processing and analysis in java; http://imagej.nih.gov/ij/index.htmls). The numbers that appear below selected western blot panels show quantifications of protein bands as percentages to the value of internal controls within the same blot. Densitometry analysis results are shown in some Figures as bar charts whose values appear relative to the mean intensity of the controls (arbitrary value 1). Densitometry comparisons were only made between bands within the same membrane and not between bands that corresponded to different blots or antibodies, unless internal controls in each blot were equalized.

### Determination of total plant protein content in leaf disks

Total proteins in leaf disks were obtained from the same leaf disk extracts that were used for the western blot analysis. Because of the incompatibility between the 1% β-mercaptoethanol in the extraction buffer and the method used in total protein quantification, proteins in those extracts were first precipitated by adding 4 volumes of cold (-20°C) acetone, followed by centrifugation for 10 minutes at 4°C at 15,000 g and pellet resuspension in 50 mM Tris pH 8.0 with 1% SDS buffer. Protein content was then measured by a Lowry-like colorimetric assay performed in microplates with DC Protein Assay (Bio-Rad, Hercules, CA, USA) using as standards known amounts of bovine serum albumin (BSA). Absorbances were read at 750 nm in a Versamax microplate reader (Molecular Devices, Sunnyvale, CA, USA).

### Quantitative RT-PCR determination of viral genomic RNA levels

For the estimation of genomic viral RNA levels in infected plants total RNAs were extracted from 3 combined leaf disks of 9.7 mm diameter taken at 7 days post-inoculation (dpi) from the same expanding analysed for viral proteins by SDS-PAGE and western blot. TRIzol reagent (Invitrogen, Carlsbad, CA, USA) was used to extract total RNA, and DNA contaminants were removed by treatment with TURBO DNA-free kit (Ambion, Austin, TX, USA). A one-step real-time quantitative reverse transcription (RT-qPCR) was performed using 15 μl of a reaction mix that contained 7.5 μl of Brilliant III Ultra-Fast RT-qPCR Master Mix (Agilent, Santa Clara, CA, USA), 1.8 μl of RNase-free water, 0.75 μl of reverse transcriptase (Agilent), 0.15 μl of 100 mM dithiothreitol (Agilent), 0.3 μM each primer, and 3 μl of total RNA extract (approximately 10 ng RNA/μl). Primers employed were PVX-Fw (5´-ATTTGGGACCAGCAACAGAG-3´) and PVX-Rv (5´-ATGCTGATTTCGGTGACTCC-3´) for PVX [30]; PVY-Fw (5´-CTGTGGGGACAAAGGGAGTA-3´) and PVY-Rv (5´-GGATGCTTGCGGATTTCATA-3´) for PVY; CMV RNA3-Fw (5´-CTGATCTGGGCGACAAGGA-3´) and CMVRNA3-Rv (5´-CGATAACGACAGCAAAACAC-3´) for RNA 3 of CMV [31]; and 18SrRNA-Fw (5´-GCCCGTTGCTGCGATGATTC-3´) and 18SrRNA-Rv (5´-GCTGCCTTCCTTGGATGTGG-3´) for 18S rRNA for normalization [30]. For the estimation of transient *GFP* transcript levels by RT-qPCR in agroinfiltrated patches for each time-point and sample in the time-course assay shown in S1 Fig, total RNAs were extracted from either two 1.5 cm diameter disks (A in S1 Fig) or from six 9.7 mm diameter leaf disks (B in S1 Fig), each from a different plant. The primers used were GFP-Fw (5´-GATGGCCCTGTCCTTTTACC-3´) and GFP-Rv (5´-CTCTCTTTTCGTTGGGATCTTTC-3´). All RT-qPCR assays were performed in a Rotor-Gene Q thermal cycler (Qiagen. Venlo, Limburgh, Netherlands) using the following protocol: 50°C for 10 min; 95°C for 3 min; 40 cycles of 95°C for 10 s and 60°C for 20 s; and a final ramp for melting analysis from 60°C to 95°C rising 1°C every 5 s. All reactions were done in triplicate with two replicates of each sample in each run. Relative viral accumulation was determined for individual plants using the 2^−ΔΔC^_T_ method with Rotor-Gene Q Series Software (Qiagen). Results were analyzed with SPSS Statistics (IBM, Armonk, NY, USA).

## Results

To assess how temperature or elevated CO_2_ levels affect compatible plant-RNA virus interactions and gene silencing suppression, we altered each of the two environmental parameters separately: 25 vs. 30°C at st CO_2_ levels, or st vs. elevated CO_2_ levels at 25°C. We infected the compatible host *N*. *benthamiana* with CMV, PVY, or a PVX vector, and the latter either unaltered or expressing the CMV 2b protein or PVY HCPro suppressors. Then we quantified systemic viral titers (CP or genomic RNA levels). We also quantified their suppressor of silencing strength in a virus-free system (agropatch assay) under those environmental conditions.

### Differential effects of temperature, CO_2_ levels on viral titers and infection symptoms are specific to each virus

**Fig 1:**
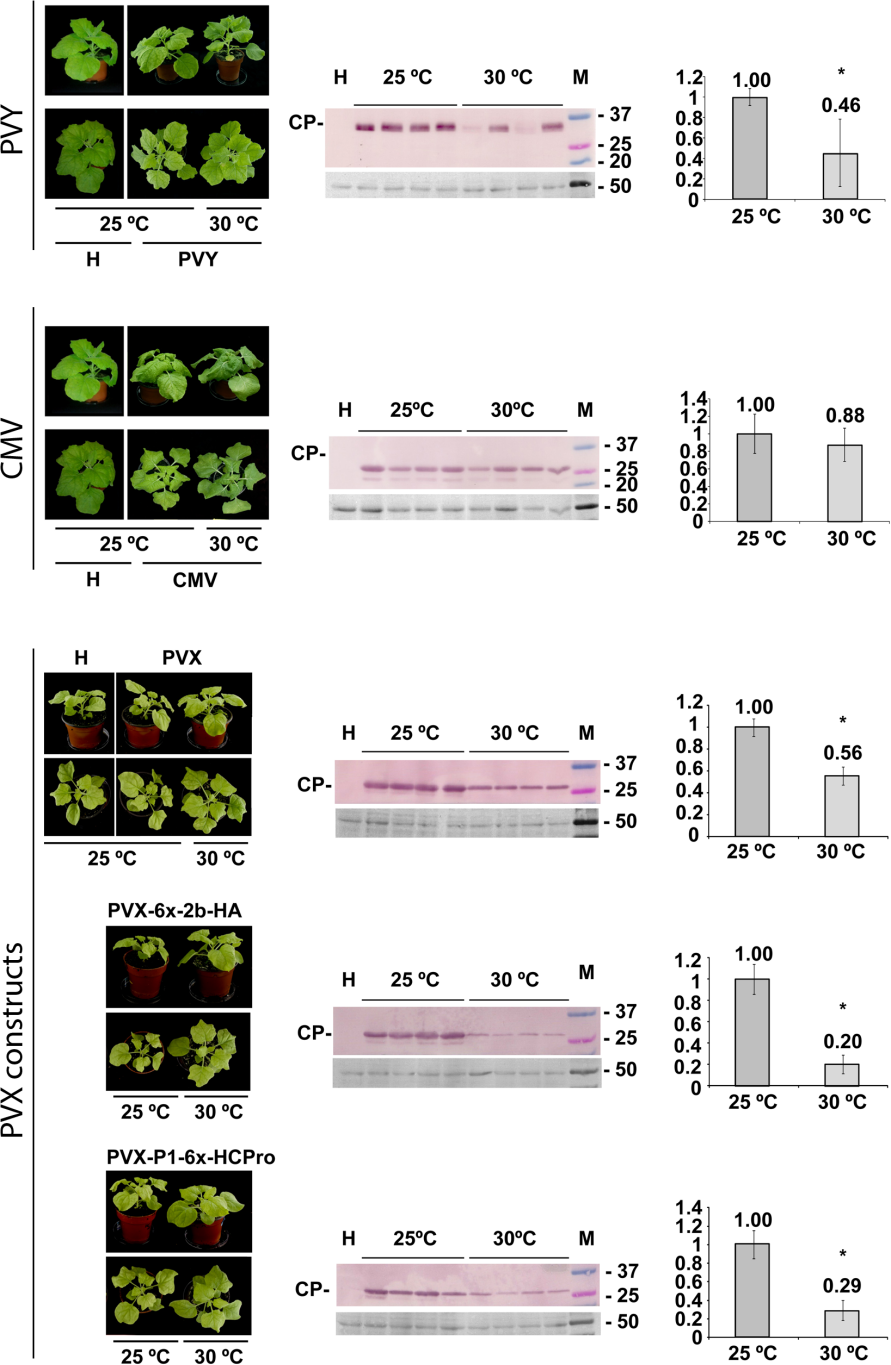
Systemic infection of *Nicotiana benthamiana* plants, at either 25°C or 30°C, by *Potato virus Y* (PVY), *Cucumber mosaic virus* (CMV), or a *Potato virus X* (PVX) vector, the latter either unaltered or expressing the CMV 2b protein or the PVY P1-HCPro bicistron (upper, middle and the three lower sets of data, respectively). Whole-plant panels in the left: at seven days post inoculation (dpi), all plants displayed infection symptoms at 25°C (mosaic, curling and stunting in the case of PVY; stronger mosaic, severe curling and stunting in the case of CMV; mosaic, curling but no stunting in all three PVX constructs, more severe in those expressing 2b or HCPro). By contrast, at 30°C only plants infected with CMV displayed symptoms similar to those observed at 25°C, whereas plants infected with PVY had very mild mosaic symptoms and plants infected with any of the three PVX vectors showed no symptoms. Plants labeled H are healthy plants. The central coat protein (CP) western blot panels assess viral titers in emerging systemic tissue: levels remained comparable at both temperatures in the case of CMV in equivalent leaf disks, but decreased markedly at 30°C in the cases of PVY and the three PVX constructs. Each western blot lane represents an extract from a single plant. Lanes labeled H show extracts from healthy plants as negative control. Lanes labeled M show molecular weight markers in kilodalton (kDa), indicated to the right of the blots. The panels below each western blot show the blotted, ponceau S-stained membranes prior to antibody incubation as controls for loading. Charts to the right of the western blot panels display mean CP values obtained in the densitometry analysis of the corresponding CP bands with standard deviation bars, relative to average CP accumulation at 25°C, which was given an arbitrary value of 1. Asterisks in charts indicate significant differences (Mann Whitney U-test, P<0.05).

**Fig 2:**
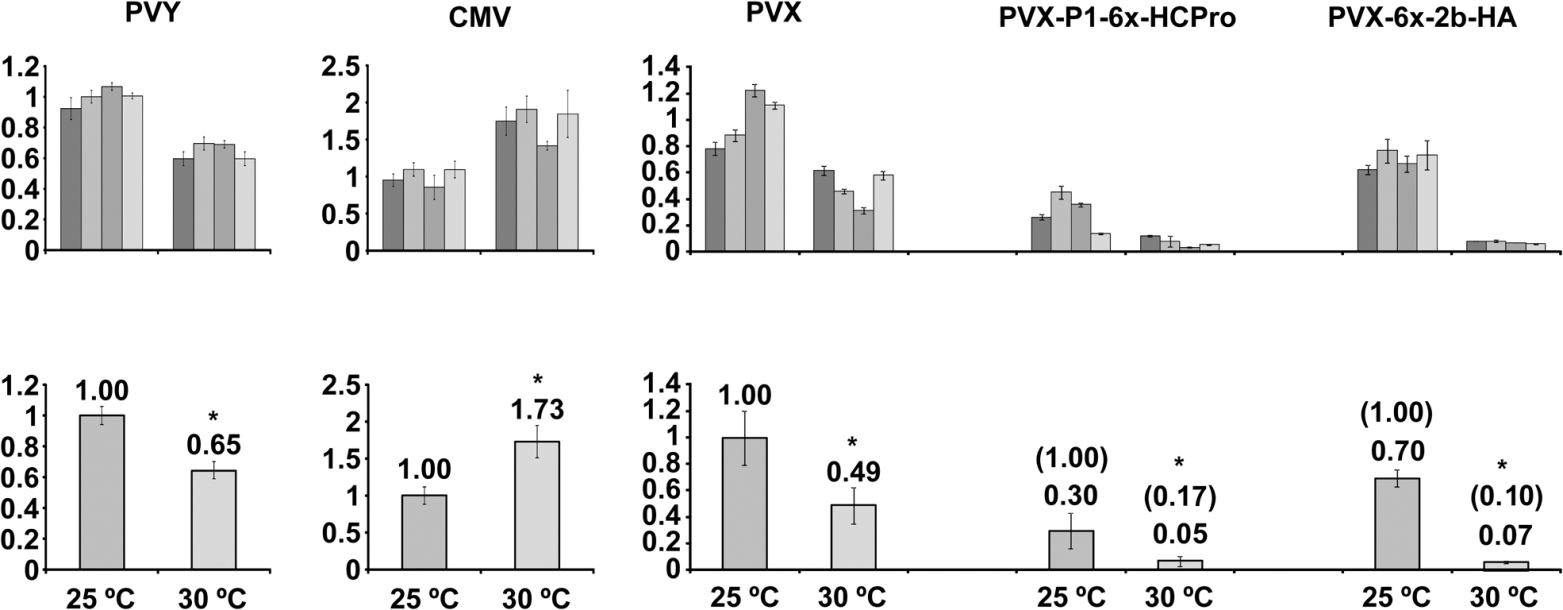
Assessment by RT-qPCR of genomic viral RNA levels in systemic tissue of *Nicotiana benthamiana* plants infected at either 25°C or at 30°C and standard (st; 401) parts per million (ppm) CO_2_ levels with *Potato virus Y* (PVY), *Cucumber mosaic virus* (CMV) or a *Potato virus X* (PVX) vector, the latter either unaltered or expressing the *Cucumber mosaic virus* (CMV) 2b protein or the PVY P1-HCPro bicistron. The upper charts show the measurements for separate individual plants, while the lower charts show the mean values with standard deviation bars, relative to viral RNA levels of PVY, CMV or the PVX constructs at 25°C, which were given an arbitrary value of 1. In the cases of the PVX vectors expressing heterologous viral suppressors, their accumulation values at 30°C relative to their accumulation at 25°C appear between brackets, while their values relative to those of PVX at 25°C, appear below. The amplified viral RNA fragments correspond to genomic RNAs, in the case of CMV to genomic RNA 3, and do not contain subgenomic sequences. Asterisks in charts indicate significant differences (Mann Whitney U-test, P<0.05).

At 30°C plants challenged with PVY or with any of the three PVX constructs showed attenuated (PVY) or no symptoms (the three PVX constructs), in contrast to infected plants kept at 25°C. By contrast, plants at 30°C challenged with CMV displayed curling and mosaic symptoms similar in severity and in their time of appearance as those in plants kept at 25°C. (Fig 1, whole plant panels on the left side). To assess systemic viral titers in individual plants, total protein extracts from leaf disks were subjected to western blot detection of viral CPs, and the intensities of individual CP bands were measured. Results showed that in the case of CMV, viral CP levels remained comparable at 25 and at 30°C. However, in the cases of PVY or of the three PVX constructs, CP levels decreased at 30°C, with regard to the levels found at 25°C (Fig 1, central western blot panels). Presence of heterologous suppressors of silencing in the PVX vectors did not attenuate the rate of reduction in the accumulation of the corresponding constructs (Fig 1). To contrast our CP data with viral genomic RNA data we measured by RT-qPCR the levels of viral RNAs (PVY and PVX genomic RNAs, CMV genomic RNA 3) extracted from leaf disks, at 25 vs. 30°C. The results obtained showed an increase of CMV levels, whereas genomic RNAs decreased for PVY and for the three PVX vectors (Fig 2 vs. Fig 1). The trends in viral RNA accumulation broadly correlated with those from CP western blots, although the larger than expected increase in CMV RNA 3 could suggest some slowing of translation or encapsidation at the higher temperature. Thus, although either of both, the CP or the viral RNA quantitation approaches seemed adequate to assess the levels of these five viruses in this host, the serological approach appears better suited for comparisons between our five different viruses.

**Fig 3:**
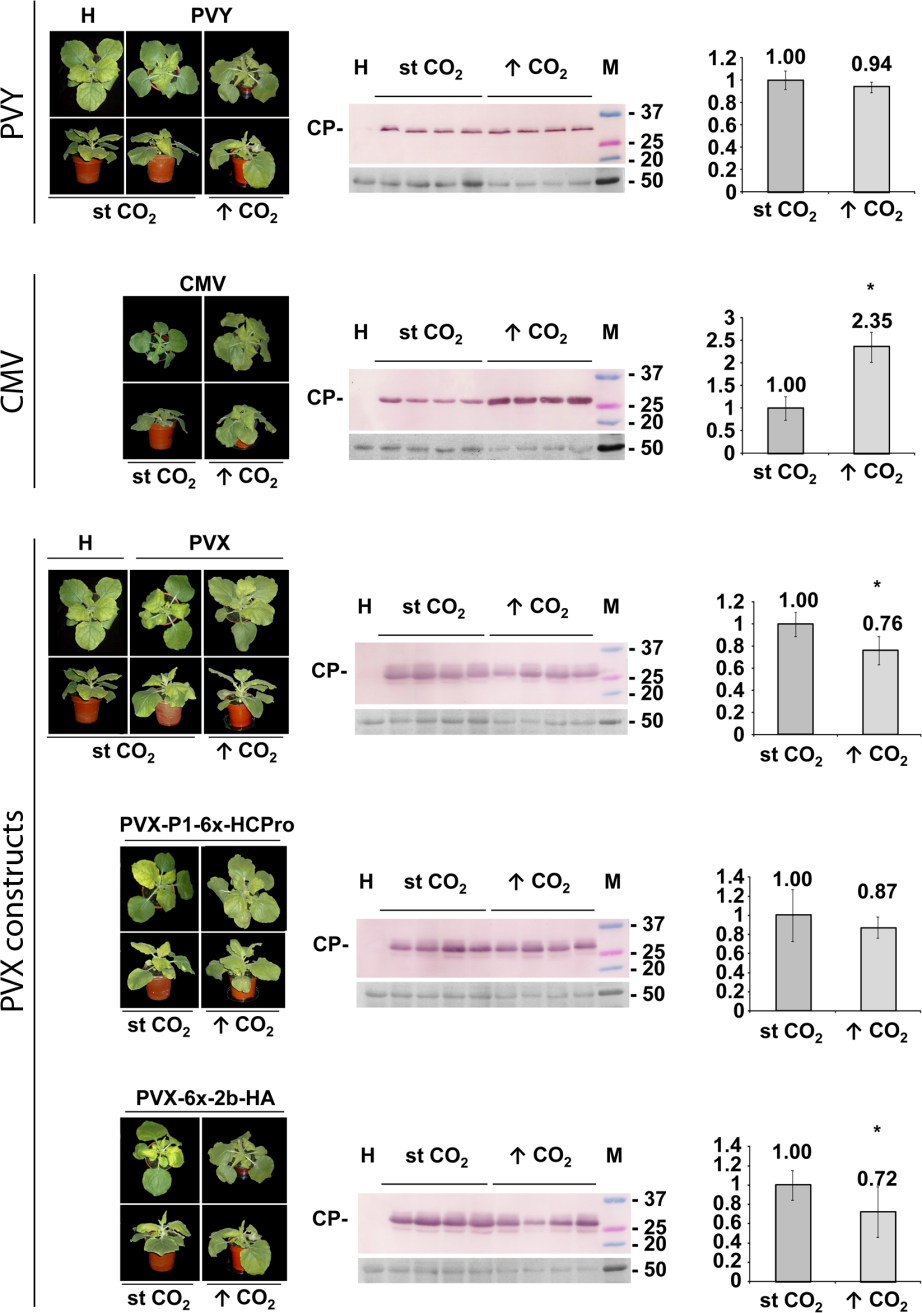
Systemic infection of *Nicotiana benthamiana* plants either at standard (st) or at elevated ↑CO_2_ levels [401 and 970 parts per million (ppm), respectively], by *Potato virus Y* (PVY), *Cucumber mosaic virus* (CMV), or a *Potato virus X* (PVX) vector, the latter either unaltered or expressing the CMV 2b protein or the PVY P1-HCPro bicistron (upper, middle and the three lower sets of data, respectively). Whole-plants in the left panels at seven days post inoculation (dpi) showed similar infection symptoms under both CO_2_ conditions (mosaic and some curling and stunting in the case of PVY; stronger mosaic, with severe curling and stunting in the case of CMV; mosaic and leaf curling but little stunting in all three PVX vectors, with more severity in those expressing heterologous suppressors of silencing 2b or HCPro). Plant panels labeled H show healthy control plants. The central coat protein (CP) western blot panels assess viral titers in emerging systemic tissue: ↑CO_2_ levels increased markedly viral titers for CMV in equivalent leaf disks, but not for PVY or the three PVX constructs. Each western blot lane represents an extract from a single plant. Lanes labeled H show an extract from a healthy plant as negative control. Lanes labeled M show molecular weight markers, indicated to the right of the blots. The panels below each western blot show the blotted, ponceau S-stained membranes prior to antibody incubation as controls for loading. Charts to the right of the western blot panels display the mean values obtained in the densitometry analysis of CP bands, with standard deviation bars relative to average CP accumulation at st CO_2_, which was given an arbitrary value of 1. Asterisks in charts indicate significant differences (Mann Whitney U-test, P<0.05).

We also challenged *N*. *benthamiana* plants with each of the five viruses, at st vs. elevated CO_2_ levels, in both cases at 25°C. Plants at elevated CO_2_ levels grew larger than those kept at st levels, but infection symptoms remained unchanged for all five viruses (Fig 3, whole plant panels to the left). Viral titers assessed by western blot analysis of viral CPs in equivalent leaf disks showed that CMV levels increased, but PVY and PVX levels fared less well than CMV at higher CO_2_ levels. However, we also noticed that the major ribulose-1,5-bisphosphate carboxylase/oxygenase (rubisco) band in the loading controls also appeared reduced in disk extracts of plants kept at high vs. st CO_2_ levels (Fig 3, ponceau S-stained loading control panels below each of the central western blot panels). We had not observed this difference in the loading control panels of extracts obtained from plants kept at different temperatures, shown in Fig 1.

### Elevated CO_2_ levels, but not high temperature, reduce total protein content in both, healthy or virus-infected *N*. *benthamiana* leaves

**Fig 4:**
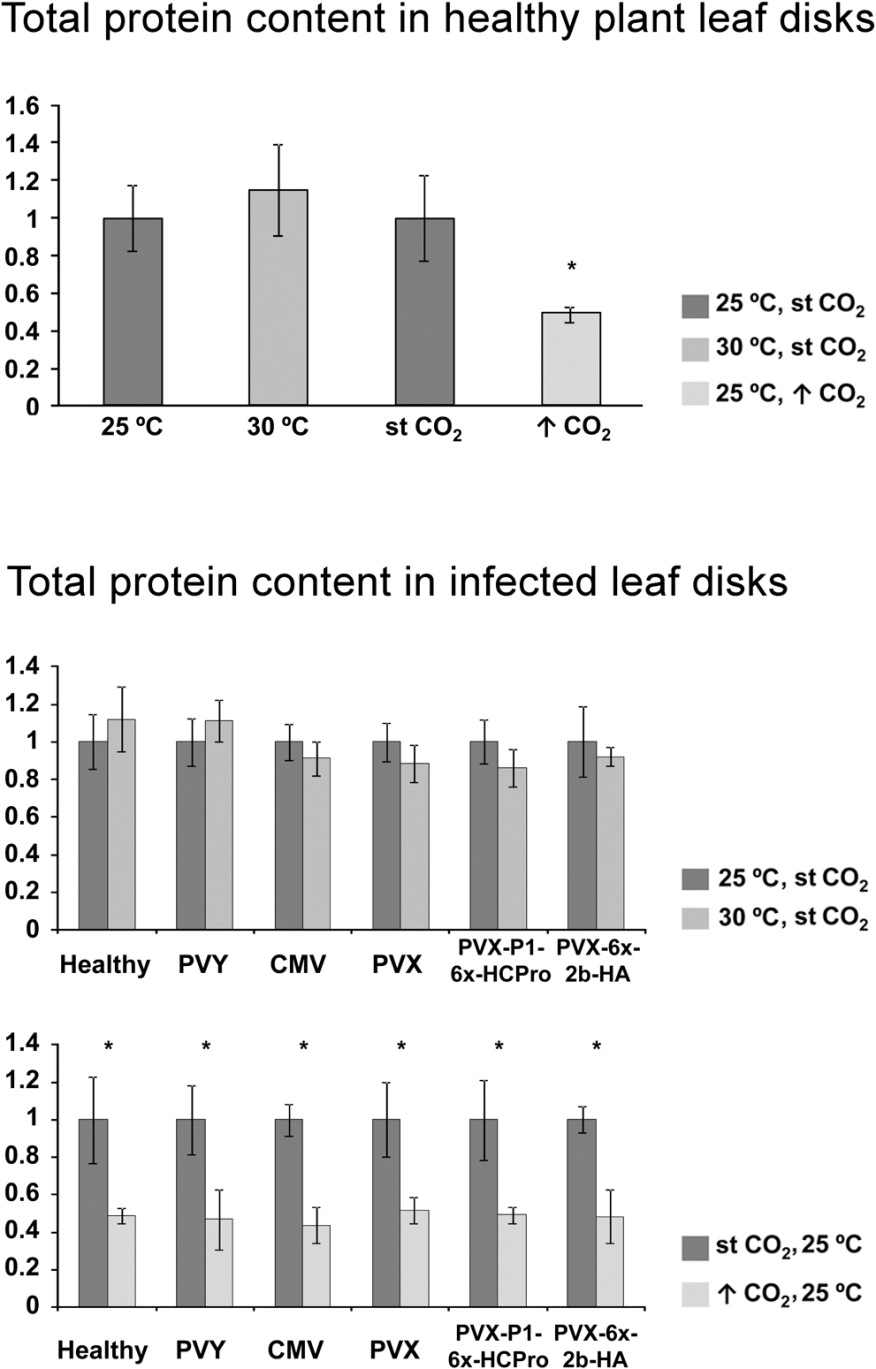
Quantification of total protein content in systemic leaf disks of *Nicotiana benthamiana* plants kept at either 25°C or at 30°C, or in plants kept at standard (st) or elevated ↑CO_2_ levels [401 and 970 parts per million (ppm), respectively]. Plants were either healthy non-inoculated, or inoculated with *Potato virus Y* (PVY), *Cucumber mosaic virus* (CMV), or a *Potato virus X* (PVX) vector, the latter either unaltered or expressing the CMV 2b protein or the PVY P1-HCPro bicistron. Results show that total protein content decreased significantly at ↑CO_2_ levels (Mann Whitney U-test, P<0.05, marked with asterisk). The upper chart shows the values for non-infected healthy plants, under the three ambient scenarios studied. The lower charts show the comparative total protein content of disks from healthy and from virus-infected plants. Total protein from leaf disks was extracted in all cases at seven days after challenge with viruses. An arbitrary value of 1 was given to total protein content in control conditions [25°C and st CO_2_ levels of 401 parts per million].

**Fig 5:**
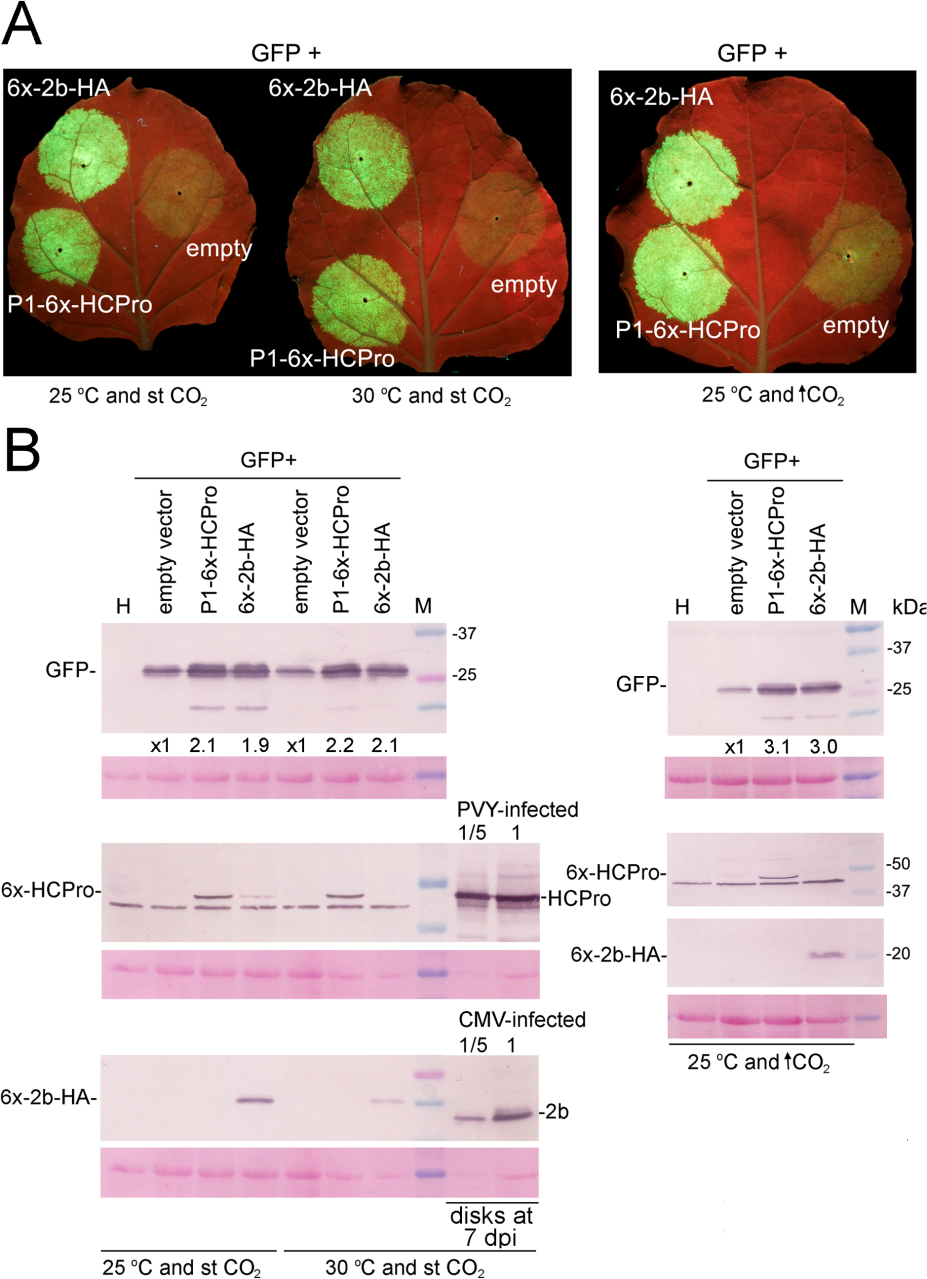
Assessment of the efficiency of the *Cucumber mosaic virus* (CMV) 2b protein or the *Potato virus Y* (PVY) P1-HCPro to alleviate the partial silencing of a green fluorescent protein (GFP) reporter under the three scenarios studied, at 72 hours post infiltration (hpi) in a virus-free system (agropatch assay). The binary vector expressing the GFP was co-infiltrated together with either an empty binary plasmid or together with plasmids expressing P1-6x-HCPro or 6x-2b-HA suppressors of silencing. **A,** visualization of GFP-derived fluorescence under the UV lamp from leaves kept at 25°C and standard (st; -401 part per million, ppm) CO_2_ levels during the 72 hpi (left side leaf), in a leaf kept the first 24 hpi at 25°C and the following 48 hpi at 30°C at st CO_2_ levels (central leaf), and from leaves kept at 25°C and elevated (↑; 970 ppm) CO_2_ levels (right side leaf). **B,** western blot analysis of the accumulation in infiltrated patches of the GFP reporter (upper panels) and of the 6x-HCPro and 6x-2b-HA protein suppressors (lower panels) using antibodies against GFP, HCPro (1A11) and the 2b protein, respectively. Below selected lanes appear the values of densitometry analysis of GFP bands (steady-state levels of GFP in control patches infiltrated with the GFP + empty binary vectors are given the arbitrary value of 1). In the high temperature scenario, the accumulation of native HCPro and of 2b protein found in equivalent-size disks from virus-infected plants at 7 days post infiltration (dpi) are also shown, either undiluted or diluted five-fold, to compare their levels with those reached by 6x-HCPro and by 6x-2b-HA proteins at 3 dpi in the agropatch assays. Lanes labeled H correspond to plant extracts from non-infiltrated plants. Lanes labeled M show molecular weight markers in kilodalton (kDa). The panels below each western blot show the blotted, ponceau S-stained membranes prior to antibody incubation as controls for loading. Note that in the ↑CO_2_ scenario only one ponceau S-stained blot appears because the same blotted membrane was cut in two separate strips for detection of both, HCPro and 2b protein, as they run at different heights.

To quantify how total plant protein content changed at elevated vs. st CO_2_ levels, or at 25 vs 30°C in *N*. *benthamiana*, and also whether viral infection might have any effect in it, total plant protein extracts used for western blots were analyzed by the Lowry method to quantify total protein amounts. The results showed that elevated CO_2_ levels alone significantly decreased total protein contents in *N*. *benthamiana* leaf disks in healthy plants (Upper chart in Fig 4), as well as in plants infected with any of the five viruses tested (lower charts in Fig 4), and virus infection did not alter this outcome at 7 dpi. When normalizing viral accumulation to total protein content instead of to leaf disks, viral accumulation as a proportion of plant protein in leaf disks were at least maintained or increased in the high CO_2_ environment, as shown in Fig 5, which summarizes our results on viral accumulation.

### Elevated temperature or CO_2_ levels do not prevent the neutralization of silencing of a reporter by viral suppressors in agropatch assays

To assess whether the viral suppressors of silencing encoded by the viruses used in this study would be capable of counteracting efficiently the silencing defenses of our compatible host *N*. *benthamiana*, under scenarios of high temperature or CO_2_ levels, we expressed transiently by agroinfiltration a GFP reporter from a binary vector, together with either an empty binary vector as control, or with binaries expressing the corresponding viral suppressors, in leaf patches. The degrees of attenuation by the suppressors of the partial silencing of the reporter could then be measured and quantified. In the case of elevated temperature, as agrobacterium-mediated delivery of T-DNAs into plant cells is prevented at temperatures above 28°C we provided a window of 24 h at 25°C to allow for efficient T-DNA transfer to take place, before rising the temperature to 30°C [16].

Both, transiently expressed constructs derived from CMV 2b protein (6x-2b-HA) and PVY HCPro (P1-6x-HCPro) increased steady-state levels of the GFP reporter three days after infiltration, at 25°C and st CO_2_ levels. The rate of increase in the accumulation of GFP induced by the suppressors was similar at 30°C than at 25°C, at st CO_2_ levels (Fig 5, left leaves and western blot panels, and reference [16]) and even higher at 25°C and high CO_2_ levels (Fig 5, right leaf and western blot panels). Therefore, under either altered parameter, the viral suppressors of silencing were capable to overcome the host silencing on the reporter. In this agropatch assays the levels reached by HCPro at 72 hpi were comparable, but not those of the 2b protein, which were lower at 30°C, suggesting a much faster turnover for the latter (Fig 5). Even so, at high temperature transient levels of 6x-2b-HA protein or of 6x-HCPro at 3 dpi in the infiltrated patches were lower than those of 2b and HCPro found in issue systemically infected by CMV or PVY virus under the same environment conditions at 7 dpi (Fig 5, lanes in left western blot panels detecting HCPro and 2b protein from virus-infected leaf disks, vs. levels of the corresponding proteins in agropatch assay disks). In a similar way as the 2b protein and HCPro, the P25 from PVX, considered a weak suppressor of silencing also increased the levels of reporter in comparable rates at 25 and at 30°C (data not shown; Aguilar *et al*., manuscript submitted).

Time-course analysis of *GFP* mRNA levels by RT-qPCR showed that in agropatch assays at 25°C the levels of *GFP* transcripts increased markedly in the presence of the 2b or HCPro suppressors (S1 Fig), whereas in assays at 30°C (the first 24 h at 25°C) transcripts also increased several-fold, but less so than at 25°C (more than 3 times in the acse of HCPro and around 2 times in the case of the 2b protein; S1 Fig), perhaps as a consequence of the interruption of T-DNA delivery into plants cells after the initial 24 hpi at 25°C.

**Fig 6:**
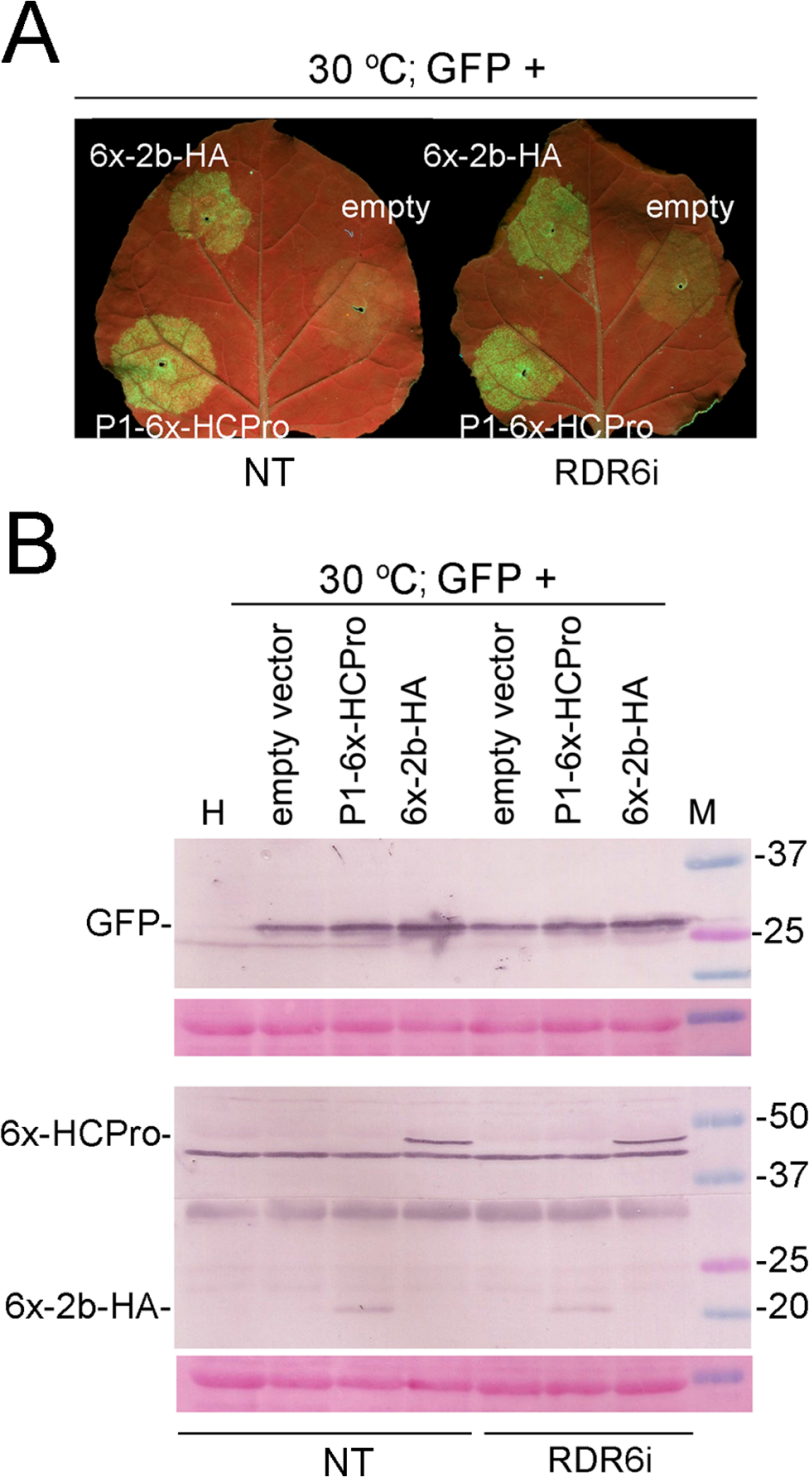
Assessment of the efficiency of the *Cucumber mosaic virus* (CMV) 2b protein or the *Potato virus Y* (PVY) P1-HCPro to release the partial silencing of a green fluorescent protein (GFP) reporter in a virus-free system (agropatch assay) in RNA-dependent RNA polymerase 6-silenced transgenic plants (line RDR6i) vs. non-transgenic (NT) plants at 30°C. Infiltrated plants were kept the first 24 hours post infiltration (hpi) at 25°C and the following 70 hpi at 30°C, at standard (st) CO_2_ levels. The GFP reporter binary vector was co-infiltrated together with either an empty binary plasmid or together with plasmids expressing P1-6x-HCPro or 6x-2b-HA suppressors of silencing. **A,** visualization of GFP-derived fluorescence under the UV lamp from infiltrated patches in NT or RDR6i leaves. **B,** western blot analysis of the accumulation in infiltrated patches of the GFP reporter (upper panels) and of the 6x-HCPro and 6x-2b-HA protein suppressors (lower panels) using antibodies against GFP, HCPro (antibody 1A11) and the 2b protein, respectively. Lanes labeled H correspond to plant extracts from non-infiltrated plants. Lanes labeled M show molecular weight markers in kilodalton (kDa). The panels below each western blot show the blotted, ponceau S-stained membranes prior to antibody incubation as controls for loading. Only one ponceau S-stained blot appears because the same blotted membrane was cut in two separate strips for detection of both, HCPro and 2b protein, as they run at different heights.

We also used RNA-dependent RNA polymerase 6-silenced plants (RDR6i) to test whether generation of RDR6-dependent secondary siRNAs influenced the levels of reporter in our sense T-DNA agropatch test. However, the levels of reporter and of viral suppressors were roughly comparable at 70 hpi to those found in non-silenced plants (Fig 6).

## Discussion

We have compared infection by five different ssRNA+ viruses of *N*. *benthamiana* plants at 25 vs. 30°C, or at st vs. elevated CO_2_ levels, and explored the relation of the infection outcome to antiviral silencing and its suppression. Systemic infections with our five RNA viruses were measured at 7 dpi in newly expanding tissue to study the initial wave of systemic infection and avoid any recovery phenotypes that could difficult our comparisons. In the three scenarios studied all plants challenged with viruses became systemically infected. However, there were stark differences with regard to severity of symptoms and virus titers. We found that at high temperature, infection by PVY resulted in very mild symptoms and reduced titers. By contrast, neither symptoms nor titers were affected in the case of Fny CMV (Figs 1 and 2). Infection by the PVX vectors followed the pattern of PVY, and the expression from the vector of the viral suppressors from CMV or PVY did not alter the disappearance of symptoms or the degree of reduction in viral titers at elevated temperature (Figs 1 and 2) suggesting that constrained PVX accumulation at high temperature was not solely caused by gene silencing or its suppression. In contrast to temperature, the effects of elevated CO_2_ on infection by these five viruses were more subtle. Plants had been kept for the previous seven days before viral challenge in elevated CO_2_ environments to adapt physiologically. We found that infection symptoms did not change with regard to plants at st CO_2_ levels, but viral titers did, in particular when measuring CP levels relative to total plant protein content in leaf disks. This was because the latter decreased always markedly under elevated CO_2_ (Fig 4 and Table 1).

**Table 1:** Summary of the effects of high temperature (30 vs. 25°C) or elevated CO_2_ levels [↑; 970 vs 401 parts per million (ppm)] on viral titers in *Nicotiana benthamiana* plants infected with *Potato virus Y* (PVY), *Cucumber mosaic virus* (CMV), or a *Potato virus X* (PVX) vector, either unaltered or expressing the CMV 2b protein or the PVY P1-HCPro bicistron. Viral titers in leaf disks or relative to total protein in leaf disks are indicated as a proportion of the values reached at 25°C or at standard (st; 401 ppm) CO_2_ levels (arbitrary values of 1), as well as are the symptoms caused to the plants.

Effect of high temperature (30°C vs. 25°C)			
**Virus**	**viral titer (25**°**C value = 1)**		**Symptoms**
	**leaf disk**	**total protein**	
PVY	0,46	0,41	attenuated
CMV	0,88	0,96	similar
PVX	0,56	0,63	symptomless
PVX-P1-6x-HCPro	0,20	0,23	symptomless
PVX-6x-2b-HA	0,29	0,31	symptomless
Effect of ↑ CO_2_ (970 ppm vs. 401 ppm)			
**Virus**	**viral titer (401 ppm value = 1)**		**Symptoms**
	**leaf disk**	**total protein**	
PVY	0,94	1,51	similar
CMV	2,35	4,57	similar
PVX	0,76	1,65	similar
PVX-P1-6x-HCPro	0,87	1,64	similar
PVX-6x-2b-HA	0,72	1,48	similar

As mentioned in the Introduction, high temperatures often lead to much weaker infection symptoms. This fact has been taken to advantage to transform plants with viral amplicons avoiding negative effects on plant regeneration and growth [32]. Coinciding with heat masking and lower viral titers, antiviral silencing was reported to increase in strength with temperature in some infections, with increases in the ratios of siRNAs to viral sequences. Thus, a causal link between heat masking and silencing strength has been proposed [10, 11, 13]. For this to occur, the effectiveness of viral suppressors in an enhanced silencing situation would also need to be somehow compromised. This is difficult to assess within an infection, as other processes operating during the viral infectious cycle could also be temperature- or CO_2_-sensitive. A way to assess silencing suppression separately from other viral processes is the use of virus-free agropatch assays, which can compare silencing suppression strength by measuring the transient steady-state levels reached by a reporter in the absence or the presence of a viral suppressor, and also with regard to the levels of suppressor [33, 34, 35, 36, 37]. Besides RNA silencing other host responses during viral infection may also be operative in agropatches: generic responses, such as non-sense mediated decay [20] or phytohormone-mediated, or case-specific ones, such as the targeted binding to viral suppressors potentially affecting their accumulation [17, 18; 19, 21, 22, 38]. A drawback of the technique is its narrow temperature scope, as agrobacterium-mediated T-DNA delivery into plants is prevented above 29°C. However this limitation can be overcome by providing a window of 24 h at permissible temperature to allow efficient T-DNA transfer before raising the temperature [16]. The amount of protein expressed from agro-delivered T-DNAs within the first 24 h, relative to the total found at the end of the assay varies for each protein [39]. In the case of our 6x-HCPro, it is undetectable serologically during the first 24 hpi [16], and thus, any of its effects on the reporter must derive largely from protein accumulating and functioning at high temperature.

To assess the biological activities of our viral suppressors we used therefore the agropatch technique. We used binary constructs that expressed a GFP reporter or the viral suppressors from the PVY and CMV isolates used in this study, modified with tags (hexahistidines fused to the N-termini of both 2b protein and HCPro, and a C-terminal hemagglutinin HA peptide fused to the 2b construct). These are also the exact same constructs expressed from the PVX vectors. This integrated study allowed us to compare the outcomes of infection by our virus isolates with the biological activities of their own suppressors under altered environment scenarios. We found that neither high temperature nor high CO_2_ levels affected negatively the ability of the suppressors to relieve the partial silencing of the GFP reporter, compared to normal conditions (25°C and st CO_2_ levels) at both, protein and transcript levels (Fig 5 and S1 Fig, respectively). *GFP* transcript accumulation in the 30°C agropatch assays had lower average levels measured at 24, 48, and 72 hpi than in the equivalent assay at 25°C. This is likely a consequence of the interruption of T-DNA delivery into plant cells after the initial 24 hpi at 25°C. We do not know why the GFP (and HCPro) product accumulated to comparable levels in both temperature assays at 72 hpi (Fig 5) despite these differences in transcript levels (S1 Fig). Perhaps lesser saturation of the translation machinery and/or higher translation rates at the higher temperature could be taking place. In any case, the presence of HCPro or the 2b protein increased transcript levels by around 3 and 2 times, respectively (S1 Fig), and these rates of increase are roughly in line with those of the GFP product shown in Fig 5.

We would expect that the same suppressor activities we found in the agropatches would operate in the context of a viral infection, as like with viruses, silencing in agropatch assays derives from plants recognizing agroinfiltration-delivered T-DNAs as foreign [33]. It could be argued that silencing at high temperature against transient T-DNA transcripts in agropatch assays could be weaker than that elicited against viruses because of insufficient secondary siRNA generation in the former. However, in RDR6i patches the levels of reporter and of viral suppressors were roughly comparable at 70 hpi to those found in non-trangenic plants (Fig 6), indicating that generation of RDR6-dependent secondary siRNAs does not determine the levels of reporter in our sense T-DNA agropatch test in the first instance. On the other hand, as transient levels of suppressor in agropatch assays were found lower than those found in virus-infected tissue (Fig 5, middle HCPro western blot panel to the left), that rules out that insufficient levels of suppressor caused the lower viral accumulation at high temperatures.

Our work therefore suggests that the lower viral titers in leaf disks that we observed for PVY and PVX in response to higher temperature or CO_2_ levels were not caused by the activities of their viral suppressors being overrun by the strength of antiviral silencing under those altered environmental conditions, and that other processes that defend plants against viruses, or other viral processes must also be involved. Viral cell-to-cell or long-distance movement appear an unlikely target, since in all five cases viruses were able to establish systemic infection at 7 dpi. Replication of positive-strand RNA viruses on the other hand is closely associated with specific virus-induced intracellular membranous vesicles [40, 41] that require translation and the translocation of viral factors via the secretory and/or cytoskeleton pathways. One possibility is that disturbations of intracellular trafficking could negatively affect our PVY and PVX isolates, but not our CMV isolate. Heat can activate endoplasmic reticulum (ER)-associated degradation pathways [41, 42] and both potyviruses and PVX, but not CMV require for their replication the formation of ER-related vesicles [43, 44, 45].

## Supporting Information

S1 Fig Quantification by RT-qPCR of *GFP* transcript levels in three-day time-course agropatch assays at either 25°C or at 30°C [the latter with the first 24 hours post infiltration (hpi) at 25°C]. Two experiments were performed. In **A**, a binary vector expressing GFP was co-infiltrated together with either empty vector or with vectors expressing the viral suppressors P1-6x-HCPro or 6x-2b-HA. Two 15 mm diameter leaf disks were collected at each of three times after infiltration (24, 48 and 72 hpi). In **B**, a GFP vector was co-infiltrated with a vector expressing β-glucuronidase (GUS; Aguilar et al. 2015. J Virol 89:2090–2103) or with vectors expressing the viral suppressors. Six 9.7 mm diameter disks were collected at each of three time points after infiltration (24, 48 and 72 hpi) from three different leaves for assessment. The rates of increase in the levels of *GFP* transcript (the combined average of the 24, 48 and 72 hpi measures) with regard to the baseline controls (either GFP + empty vector in A or GFP + GUS vector in B, both given a value of x1) are indicated below each sample.
(TIF)
