# Supplementary figures and images for: High Temperature, High Ambient CO2 Affect the Interactions between Three Positive-Sense RNA Viruses and a Compatible Host Differentially, but not Their Silencing Suppression Efficiencies
Source: PLoS One. 2015 Aug 27;10(8):e0136062. doi: 10.1371/journal.pone.0136062 (PMC4551900; doi:10.1371/journal.pone.0136062)

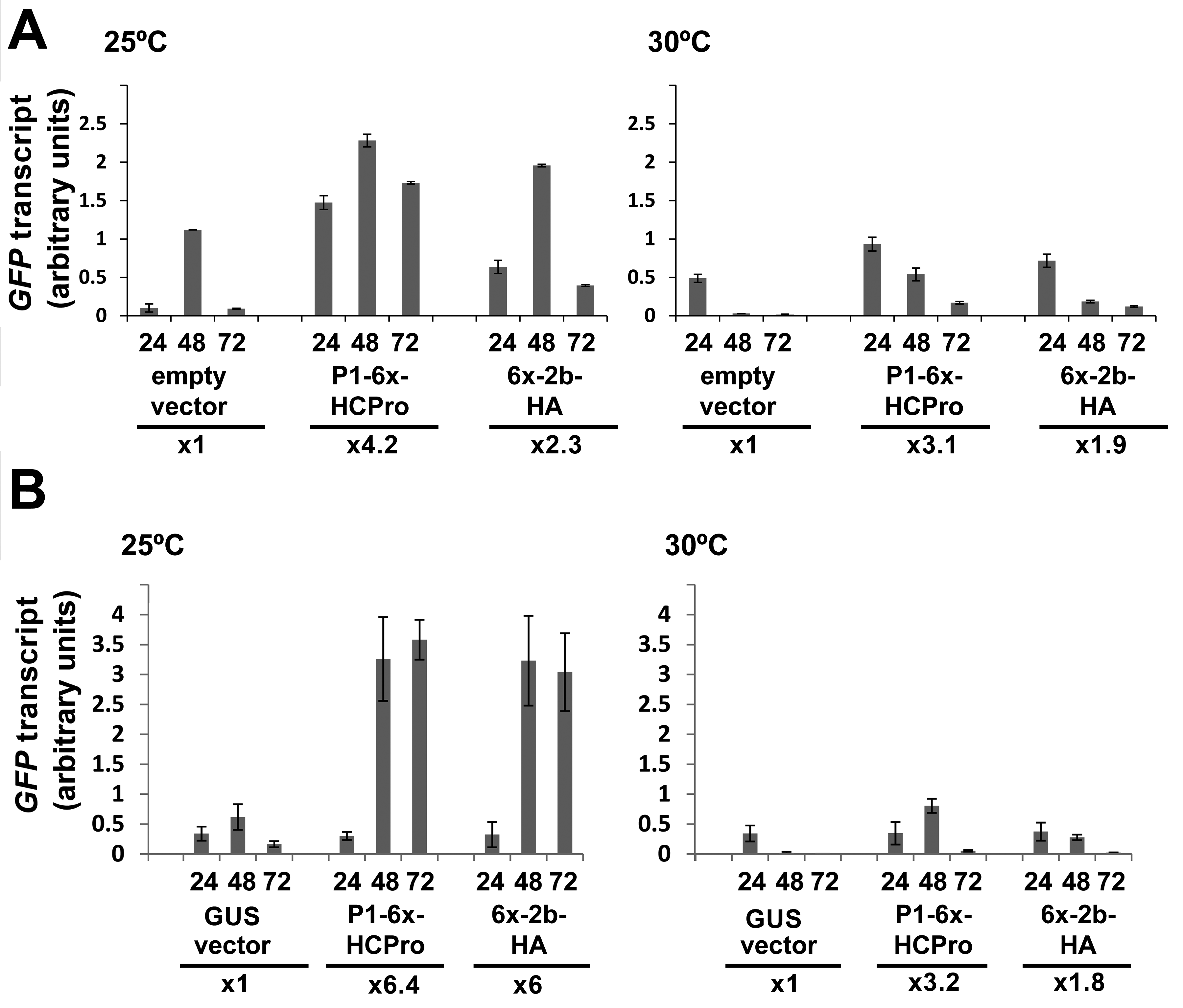

Supplement: S1 Fig — Two experiments were performed. In A, a binary vector expressing GFP was co-infiltrated together with either empty vector or with vectors expressing the viral suppressors P1-6x-HCPro or 6x-2b-HA. Two 15 mm diameter leaf disks were collected at each of three times after infiltration (24, 48 and 72 hpi). In B, a GFP vector was co-infiltrated with a vector expressing β-glucuronidase (GUS; Aguilar et al. 2015. J Virol 89:2090–2103) or with vectors expressing the viral suppressors. Six 9.7 mm diameter disks were collected at each of three time points after infiltration (24, 48 and 72 hpi) from three different leaves for assessment. The rates of increase in the levels of GFP transcript (the combined average of the 24, 48 and 72 hpi measures) with regard to the baseline controls (either GFP + empty vector in A or GFP + GUS vector in B, both given a value of x1) are indicated below each sample. (TIF) [file pone.0136062.s001.tif]
